# Intestinal ELF4 Deletion Exacerbates Alcoholic Liver Disease by Disrupting Gut Homeostasis

**DOI:** 10.3390/ijms23094825

**Published:** 2022-04-27

**Authors:** Tongtong Liu, Haitao Yu, Zeming Zhang, Yunfei Xie, Long Yang, Fuping You

**Affiliations:** 1 Institute of Systems Biomedicine, Department of Immunology, School of Basic Medical Sciences, Beijing Key Laboratory of Tumor Systems Biology, Peking University Health Science Center, Beijing 100191, China; tongtliu@pku.edu.cn (T.L.); yuhaitao@bjmu.edu.cn (H.Y.); zm_zhang@bjmu.edu.cn (Z.Z.); 1610305227@bjmu.edu.cn (Y.X.); 2 Research Center for Infectious Diseases, Tianjin University of Traditional Chinese Medicine, Tianjin 301617, China; 3 School of Integrative Medicine, Tianjin University of Traditional Chinese Medicine, Tianjin 301617, China

**Keywords:** ELF4, ALD, gut microbiota, intestinal barrier

## Abstract

Alcohol liver disease (ALD) is characterized by intestinal barrier disruption and gut dysbiosis. Dysfunction of E74-like ETS transcription factor 4 (ELF4) leads to colitis. We aimed to test the hypothesis that intestinal ELF4 plays a critical role in maintaining the normal function of intestinal barrier and gut homeostasis in a mouse model of ALD. Intestinal ELF4 deficiency resulted in dysfunction of the intestinal barrier. *Elf4^−/−^* mice exhibited gut microbiota (GM) dysbiosis with the characteristic of a larger proportion of Proteobacteria. The LPS increased in *Elf4^−/−^* mice and was the most important differential metabolite between *Elf4^−/−^* mice and WT mice. Alcohol exposure increased liver-to-body weight ratio, and hepatic inflammation response and steatosis in WT mice. These deleterious effects were exaggerated in *Elf4^−/−^* mice. Alcohol exposure significantly increased serum levels of TG, ALT, and AST in *Elf4^−/−^* mice but not in WT mice. In addition, alcohol exposure resulted in enriched expression of genes associated with cholesterol metabolism and lipid metabolism in livers from *Elf4^−/−^* mice. 16S rRNA sequencing showed a decrease abundance of *Akkermansia* and *Bilophila* in *Elf4^−/−^* mice. In conclusion, intestinal ELF4 is an important host protective factor in maintaining gut homeostasis and alleviating alcohol exposure-induced hepatic steatosis and injury.

## 1. Introduction

Alcoholic liver disease (ALD), which ranges from asymptomatic hepatic steatosis to the development of cirrhosis and hepatocellular carcinoma [1], is one of the most common causes of liver diseases worldwide [2]. ALD is a major contributor to the global burden of mortality and disease [3]. The pathogenesis of ALD involves complex factors including hepatic steatosis, toxic metabolites of alcohol metabolism, oxidative stress, pro-inflammatory cytokines, and gut microbiota (GM) dysbiosis [4,5]. Alcohol intake impairs the function of the intestinal barrier and gut homeostasis, resulting in an increased permeability [6], altered bacterial composition and increased serum lipopolysaccharide (LPS) levels [7,8]. LPS, also known as endotoxin, is derived from Gram-negative bacteria of the intestinal tract. LPS can bind to the Toll-like receptor (TLR) 4 complex on the surface of immune cells, resulting in activation of the inflammatory cascades [9].

The intestinal barrier is the physical and functional zone of interaction between the luminal microbiome and the host [10], and increased intestinal permeability contributes to pathogenesis of ALD [11]. The paracellular space in epithelial cells is sealed by tight junctions (TJs), which form a permeability barrier and act as a membrane fence [12]. Goblet cells in the intestinal epithelium secrete mucins and form the mucus layer which acts as a physical barrier [13]. Meanwhile, the increased intestinal permeability and GM dysbiosis contributes to the abnormal gut–liver axis in ALD [14,15,16].

ELF4 belongs to the E74-like factor (ELF) subfamily of the E26 transformation-specific (ETS) transcription factor family and is involved in regulating immune responses and development of immune-related cells [17]. Our recent work has shown that ELF4 knockout mice develop normally but are sensitive to dextran sulfate sodium salt (DSS) induced colitis, and azoxymethane (AOM) combined with DSS induced colitis-associated cancer (CAC) [18]. A recent report indicates that mutations of ELF4 in patients causes human autoinflammatory disease with inflammatory bowel disease (IBD) characteristics [19]. ELF4-mutant macrophages had hyperinflammatory responses to a range of innate stimuli and the deficiency of ELF4 augments inflammatory TH17 cell responses, which result in mucosal autoinflammation and IBD characteristics in patients with ELF4 loss-of-function variants [19]. Taken together, we can conclude that dysfunction of ELF4 leads to colitis in both induced mouse models and human patients [18,19]. However, the mechanism by which ELF4 protects human and mice from IBD or autoinflammatory disease is largely unknown. Enterocytes and Paneth cells can regulate gut homeostasis by producing MUCs and antimicrobial peptides [13,20], while the disruption of the intestinal barrier impairs this function [21]. Leaky gut and GM dysbiosis contribute to the pathogenesis of various common metabolic disorders including ALD [4,22,23]. Thus, it is necessary to evaluate ELF4 deficiency’s influence on the intestinal barrier and GM, and whether ELF4 knockout increases ALD susceptibility.

Here, we showed that ELF4 knockout mice exhibited increased intestinal permeability and exhibited GM dysbiosis which led to increased LPS levels. We discovered that ELF4 knockout exacerbates alcohol-induced GM dysbiosis and liver injury. Our findings identify ELF4 as an important host factor in maintaining gut homeostasis and may provide a potential strategy for preventing ALD by targeted manipulation of ELF4.

## 2. Results

### 2.1. ELF4 Deficiency Leads to Increase in Colon Permeability

**Figure 1:**
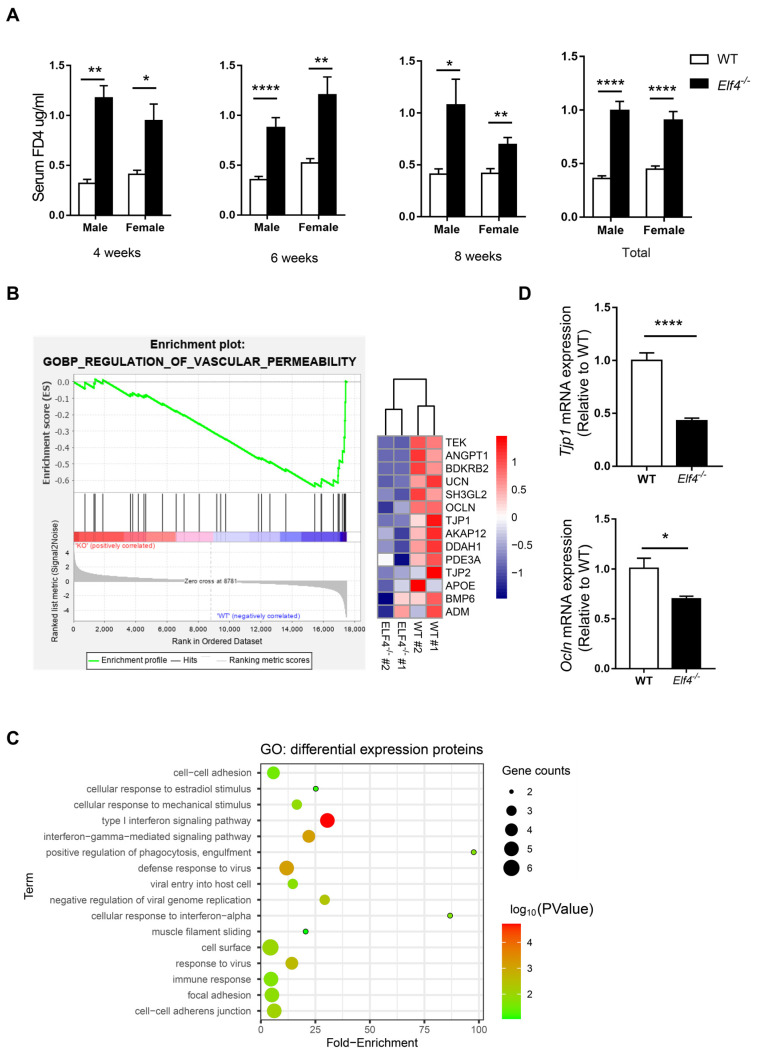
ELF4 deficiency leads to an increase in colon permeability. (**A**) Serum 4 kDa FITC-Dextran (FD4) level of indicated mice of different ages and sexes after 0.1 mg/g FD4 gavage for 3 h. Four weeks, male-WT, *n* = 2, male-*Elf4^−/−^*, *n* = 2, female-WT, *n* = 4, female-*Elf4^−/−^*, *n* = 7; 6 weeks, male-WT, *n* = 15, male-*Elf4^−/−^*, *n* = 10, female-WT, *n* = 7, female-*Elf4^−/−^*, *n* = 8; 8 weeks, male-WT, *n* = 3, male-*Elf4^−/−^*, *n* = 5, female-WT, *n* = 12, female-*Elf4^−/−^*, *n* = 13; total, male-WT, *n* = 20, male-*Elf4^−/−^*, *n* = 19, female-WT, *n* = 23, female-*Elf4^−/−^*, *n* = 28. * *p <* 0.05, ** *p <* 0.01, **** *p <* 0.0001. Unpaired two-sided Student’ s *t*-test, data are represented as mean ± SEM. (**B**) GSEA enrichment of regulation of vascular permeability pathway between ELF4^−/−^ Caco2 (clone1 and clone2 were used) and WT Caco2 cells. ‘KO’ refers to ELF4^−/−^ Caco2 (left panel). NES = −1.99. A heatmap of indicated genes in the vascular permeability pathway (right panel). (**C**) Enrichment of differential expression proteins between ELF4^−/−^ Caco2 and WT Caco2 cells by label-free proteomics. (**D**) Colon *Tjp1* (upper panel) and *Ocln* (low panel) mRNA expression of *Elf4^−/−^* mice and WT mice by RT-qPCR. WT, *n* =10, *Elf4^−/−^*, *n* = 14. * *p <* 0.05, **** *p <* 0.0001. Unpaired two-sided Student’ s *t*-test, data are represented as mean ± SEM.

Our previous study has shown that ELF4 transcriptionally regulates multiple DNA damage repair machinery components, and that ELF4 deficiency leads to cell damage and mice sensitive to DSS-induced colitis [18]. Additionally, intestinal epithelial cell injury is associated with higher intestinal permeability [24]. To identify ELF4 deficiency’s influence on the intestinal barrier, we used intestinal epithelial specific ELF4 knockout (*Elf4^−/−^*) mice which were bred from Vil1-Cre mice and Elf4^loxp/loxp^ mice (Supplementary Figure S1A) to examine gut permeability. We selected mice of different ages and sexes to detect the intestinal permeability levels. As shown in Figure 1A, the serum 4 kDa FITC-Dextran (FD4) level increased significantly in both *Elf4^−/−^* male and female mice compared with WT mice in all three age groups (*p <* 0.05, *p <* 0.01 or *p <* 0.0001 as indicated). To further explore ELF4 deficiency’s effect on permeability, we constructed ELF4^−/−^ Caco2 clones through the CRISPR-Cas9 system (Supplementary Figure S1B). The gene expressions of ELF4^−/−^ Caco2 clones and WT Caco2 cells were detected through RNA-seq. Gene set enrichment analysis (GSEA) showed that the ‘regulation of vascular permeability pathway’ was enriched in WT phenotypes (Figure 1B). A heatmap showed that tight junction associated genes including TJP1 and OCLN had decreased expression in ELF4^−/−^ Caco2 (Figure 1B). Then, we undertook a label-free proteomics to study the protein levels in ELF4^−/−^ Caco2 clones and WT Caco2 cells. We detected a total of 1797 proteins, of which 22 showed a significant difference between ELF4^−/−^ Caco2 clones and WT Caco2 cells (Supplementary Figure S1C). These differential expression proteins (DEPs) were enriched in innate immunity and virus response which is consistent with our previous study [25] (Figure 1C). Among the enriched pathways, we also observed cell–cell adhesion and focal adhesion (Figure 1C). We detected the mRNA expression of *Tjp1* and *Ocln* in the colons of *Elf4^−/−^* mice and WT mice by RT-qPCR. *Tjp1* mRNA expression significantly decreased in *Elf4^−/−^* mice compared with WT mice as well as *Ocln* mRNA expression (Figure 1D) (*p <* 0.05 or *p <* 0.0001 as indicated). Taken together, our data demonstrates that ELF4 deficiency leads to decreased expression of tight junction-related genes and higher permeability.

### 2.2. Elf4^−/−^ Mice Exhibit GM Dysbiosis

**Figure 2:**
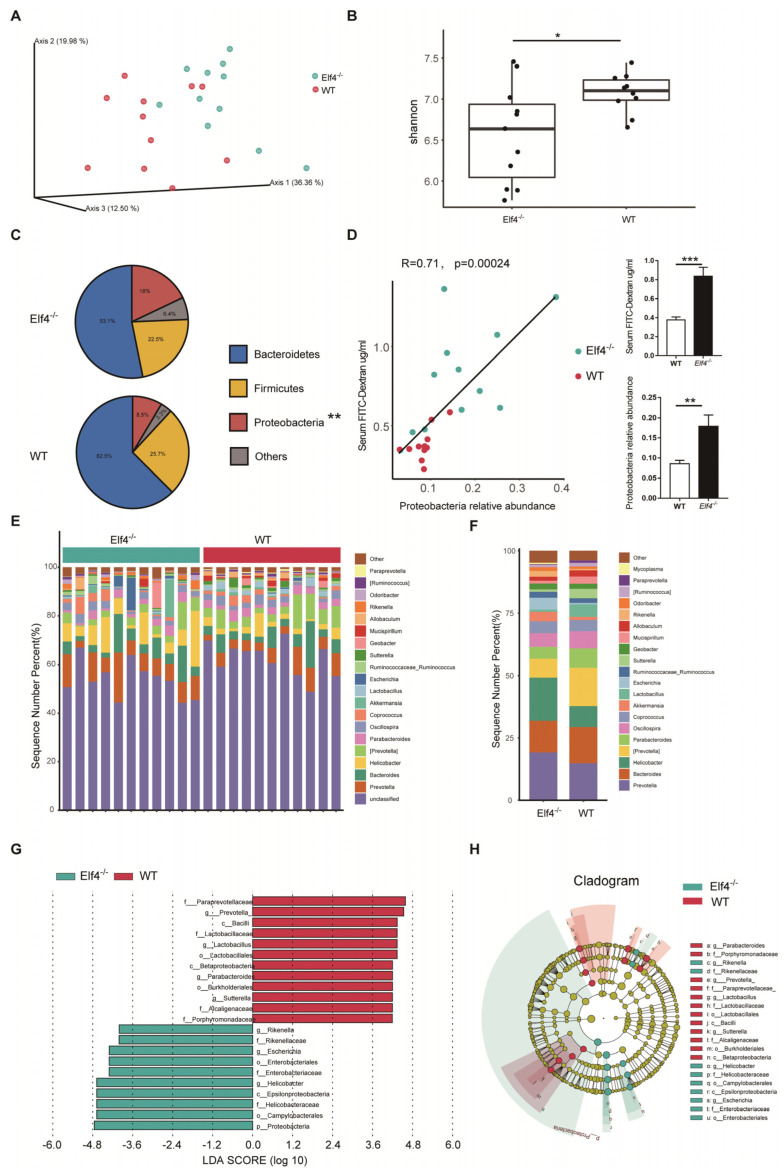
*Elf4^−/−^* mice exhibit gut microbiota dysbiosis. Feces of 6-week-old *Elf4^−/−^* mice and WT mice were taken and deep sequencing of 16s rRNA was performed. *Elf4^−/−^* mice, *n* = 11, WT mice, *n* = 11. (**A**) Principal Coordinate Analysis (PCoA) using the core-diversity plugin within QIIME2. (**B**) Shannon index calculating the alpha-diversity in *Elf4^−/−^* mice and WT mice. * *p* < 0.05, unpaired two-sided Student’ s *t*-test. (**C**) Pie charts showing the relative phylum detected in the feces of *Elf4^−/−^* mice and WT mice, top 3 phyla were shown. (**D**) Linear correlation analysis between gut permeability and fecal Proteobacteria relative abundance of WT and *Elf4^−/−^* mice (left panel). Serum FD4 level after 0.1 mg/g FD4 gavage for 3 h and fecal Proteobacteria relative abundance, between *Elf4^−/−^* mice and WT mice (right panel). ** *p* < 0.01, *** *p* < 0.001. Unpaired two-sided Student’ s *t*-test, data are represented as mean ± SEM. (**E**,**F**) Sequence number percentage of different intestinal bacteria genus of *Elf4^−/−^* mice and WT mice samples (**E**) or groups (**F**), top 20 most prevalent were shown. (**G**) Linear discriminant analysis Effect Size (LEfSe) analysis shows the differential fecal bacteria genera between *Elf4^−/−^* mice and WT mice with linear discriminant analysis (LDA) scores greater than 4. (**H**) Cladogram showed differential fecal bacteria with LDA scores greater than 4.

Normal intestine function is necessary for maintaining a healthy microbiota community [26]. Reported results indicate that increased intestinal permeability is related to fecal microbiota dysbiosis [15]. Considering that increased intestinal permeability has been observed in *Elf4^−/−^* mice, we hypothesized that intestinal ELF4 deficiency could lead to GM disorders. Deep sequencing of 16s rRNA was performed to identify the bacterial taxa associated with ELF4 knockout. We compared the fecal GM composition of *Elf4^−/−^* mice and WT mice. Principal Coordinate Analysis (PCoA) showed that GM clustered differently between *Elf4^−/−^* mice and WT mice (Figure 2A). The alpha-diversity calculated by the Shannon index showed decreased bacterial species diversity in *Elf4^−/−^* mice (Figure 2B) (*p* < 0.05). We identified that *Elf4^−/−^* mice had a specific GM with the characteristic of a larger proportion of Proteobacteria (Figure 2C). The increase in Proteobacteria which contains multiple pathogens is regarded as a sign of GM dysbiosis [27]. Moreover, there was a positive correlation between proportion of Proteobacteria and intestinal permeability in *Elf4^−/−^* mice and WT mice (Figure 2D) (*p* < 0.001 or *p* < 0.01 as indicated). As shown in Figure 2E,F, the microbiota of *Elf4^−/−^* mice and WT mice showed differential genera compositions. The linear discriminant analysis (LDA) score and cladogram, reflecting relative bacterial abundance (Figure 2G,H), showed that the *Prevotella* and the *Parabacteroides* genera of the Bacteroidetes phylum, the *Lactobacillus* genus of the Firmicutes phylum, and the *Sutterella* genus of the Proteobacteria phylum were overrepresented in WT mice. Conversely, the *Helicobacter* and the *Escherichia* genera of the Proteobacteria phylum and the *Rikenella* genus of the Bacteroidetes phylum were specifically overrepresented in *Elf4^−/−^* mice. These data show that *Elf4^−/−^* mice exhibited GM dysbiosis with an increased percentage of the Proteobacteria phylum.

### 2.3. An Increase in the Phylum Proteobacteria Is Positively Correlated with Higher LPS Levels

**Figure 3:**
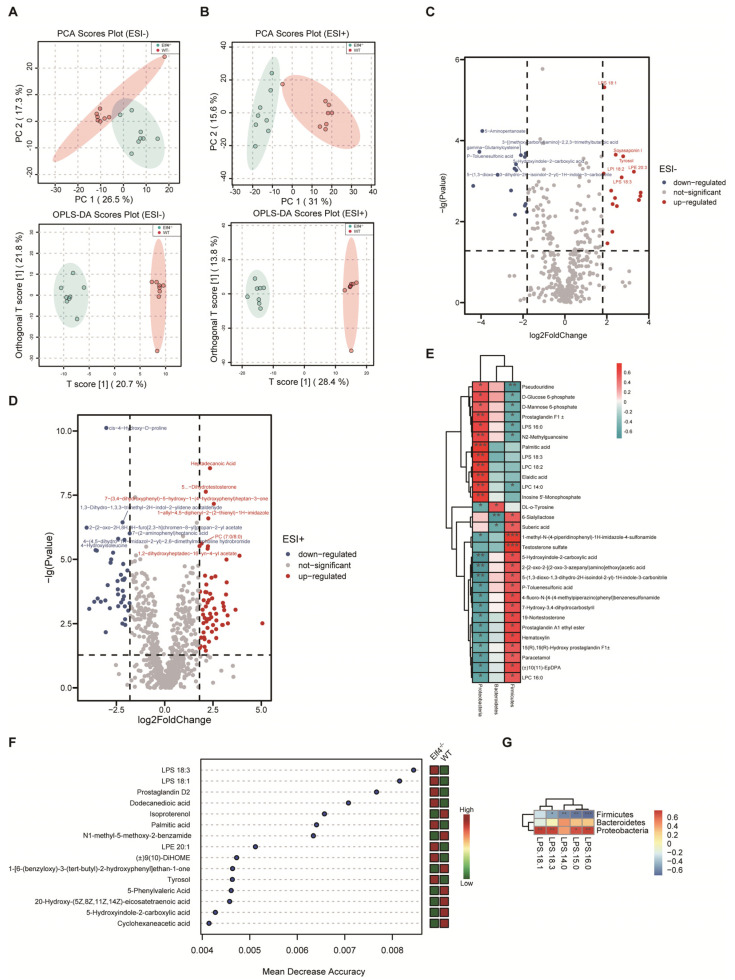
An increase in the phylum Proteobacteria is positively correlated with higher LPS levels. UHPLC-MS analysis of feces from WT mice and *Elf4^−/−^* mice. (WT mice, *n* = 9; *Elf4^−/−^* mice, *n* = 9). (**A**,**B**) PCA scores plot and OPLS-DA scores plot between the WT group and *Elf4^−/−^* group in the ESI+ model (**A**) and the ESI− model (**B**). (**C**,**D**) Volcano plots show differential metabolites in *Elf4^−/−^* mice compared with WT mice in the ESI− model (**C**) and the ESI+ model (**D**). (**E**) Heatmap shows linear correlation analysis between main fecal metabolites and the relative abundance of fecal bacteria (WT mice and *Elf4^−/−^* mice data included). (**F**) Random forest (RF) showing the most important features in the sample grouping prediction (left panel). Heatmap showing the relative abundance of these metabolites in WT mice and *Elf4^−/−^* mice (right panel). (**G**) Heatmap showing linear correlation analysis between LPS and the relative abundance of fecal bacteria (WT mice and *Elf4^−/−^* mice data included).

To explore the consequences of GM dysbiosis, we investigated the metabolic profiles of feces from *Elf4^−/−^* mice and WT mice. The total ion chromatograms (TICs) of the feces samples were analyzed by UHPLC-MS/MS and processed using the Compound Discoverer v3.1, then the R package MetaboAnalystR was used for multivariate analysis. Principle component analysis (PCA) and orthogonal partial least squares discriminatory analysis (OPLS-DA) were applied to discriminate the difference between *Elf4^−/−^* group and WT group. According to the PCA scores plot and the OPLS-DA scores plot, the *Elf4^−/−^* and WT groups were clearly separated into two clusters in ESI− (Figure 3A) and ESI+ (Figure 3B), which indicated that there was a remarkable difference in fecal metabolite profiles within the two groups. To prevent the over-fitting effects, we performed a permutation including 100 tests to validate the predictive ability. The obtained parameters R2Y (cum) and Q2 (cum) were 0.986, 0.812 in the negative ion model, and 0.997, 0.947 in the positive ion model, which indicated that the model was reliable (Supplementary Figure S2). There were 30 differential metabolites in the negative ion model and 99 differential metabolites in the positive ion model which were filtered out with *p*-value < 0.05 and log2(foldchange) > 1.8 (Figure 3C,D). Furthermore, increasing evidence uncovered that the gut microbiota can produce bioactive molecules that affect host metabolism, indirectly involved in the regulation of host metabolic states [28,29]. To explicate the relationship between these differential metabolites and the relative abundance of phyla, we undertook a linear correlation analysis. Our findings showed that those metabolites that were negatively correlated with the Proteobacteria phylum were positively correlated with the Firmicutes phylum, and those metabolites that were positively correlated with the Proteobacteria phylum were negatively correlated with the Firmicutes phylum (Figure 3E). In order to identify the most important differential metabolites between the *Elf4^−/−^* group and WT group, we conducted a random forest (RF) and found that LPS was the most important metabolite (Figure 3F). The LPS level was positively correlated with the Proteobacteria phylum and negatively correlated with the Firmicutes phylum (Figure 3G). Taken together, ELF4 exerts critical roles on the gut microbial composition and metabolic homeostasis.

### 2.4. Elf4^−/−^ Mice Showed Increased Alcohol-Induced Hepatic Inflammation Response

In the case of increased intestinal permeability, GM and their metabolites are able to enter the liver through blood vessels, bind to different receptors, affect liver metabolism, induce inflammatory responses, and result in liver injury [30]. As an important pathogenic factor, LPS can bind to TLR4 of Kupffer cells and cause liver inflammation and liver damage [31].

**Figure 4:**
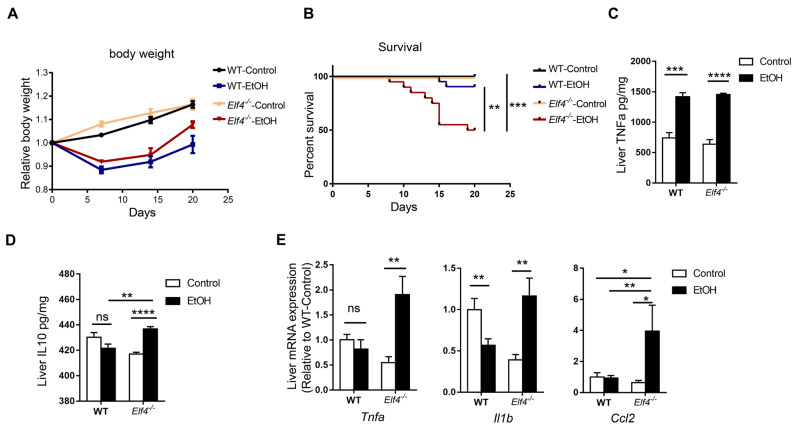
*Elf4^−/−^* mice have increased alcohol-induced hepatic inflammation response. (**A**,**B**) Body weight (**A**) and survival (**B**) of *Elf4^−/−^* mice and WT mice treated with alcohol diet or control diet for 21 days. WT-Control, *n* =18, WT-EtOH, *n* = 12, *Elf4^−/−^*-Control, *n* = 18, *Elf4^−/−^*-EtOH, *n* = 20. Data are represented as mean ± SEM. (**C**,**D**) Liver TNFa level (*** *p* < 0.001, **** *p* < 0.0001) (**C**) and IL10 level (NS, not significant) (**D**) quantified by ELISA. *n* = 5. Unpaired two-sided Student’s *t*-test, data are represented as mean ± SEM. (**E**) Quantitative real-time polymerase chain reaction (RT-qPCR) analysis of indicated liver genes between *Elf4^−/−^* mice and WT mice treated with alcohol diet or control diet for 21 days. WT-Control, *n* = 10, WT-EtOH, *n* = 14, *Elf4^−/−^*-Control, *n* = 8, *Elf4^−/−^*-EtOH, *n* = 4. NS, not significant, * *p* < 0.05; ** *p* < 0.01. Unpaired two-sided Student’s *t*-test, data are represented as mean ± SEM.

To further explore intestinal ELF4 knockout’s effect on the liver, we fed *Elf4^−/−^* mice and WT mice with the DeCarli liquid diet containing 5% (*w*/*v*) alcohol (EtOH) or isocaloric maltose dextrin (Control) for 21 days. We found that *Elf4^−/−^* mice showed more severe liver injury. Both groups of mice treated with alcohol had lower body weight (Figure 4A). Alcohol feeding decreased the survival rate of *Elf4^−/−^* mice compared with WT mice (Figure 4B) (*p* < 0.01 or *p* < 0.001 as indicated).

Alcohol feeding increased hepatic inflammation in *Elf4^−/−^* mice. The protein level of proinflammatory cytokine TNFa increased in both the WT-EtOH and the *Elf4^−/−^*-EtOH groups, compared with the control groups (Figure 4C) (*p* < 0.001 or *p* < 0.0001 as indicated). Anti-inflammatory cytokine IL10 had no difference in livers of WT mice, but significantly increased in those of *Elf4^−/−^* mice (Figure 4D) (*p* < 0.01 or *p* < 0.0001 as indicated). Furthermore, RT-qPCR analysis showed significantly elevated gene expression in the proinflammatory cytokines *Tnfa* and *Il1b*, as well as chemokine *Ccl2* in the *Elf4^−/−^*-EtOH group (Figure 4E) (*p* < 0.01 or *p* < 0.05 as indicated). These results demonstrated that *Elf4^−/−^* mice showed enhanced hepatic inflammation response upon alcohol exposure.

### 2.5. Elf4^−/−^ Mice Showed Increased Alcohol-Induced Hepatic Steatosis

**Figure 5:**
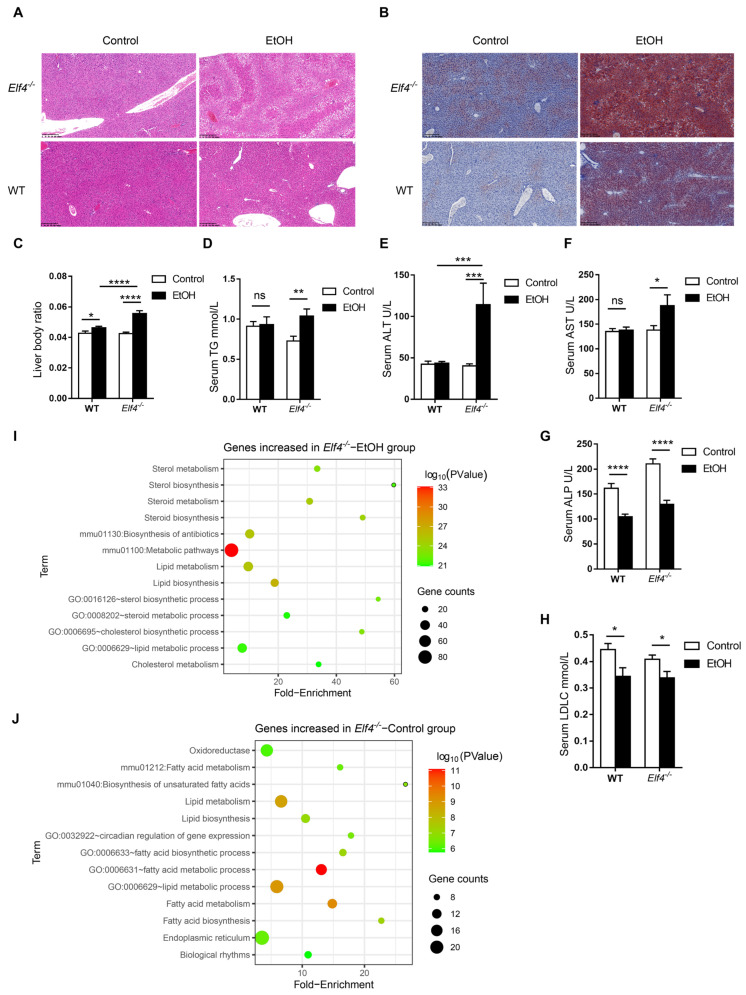
*Elf4^−/−^* mice have increased alcohol-induced hepatic steatosis. (**A**) Hematoxylin–eosin staining of the liver sections of *Elf4^−/−^* mice and WT mice treated with alcohol diet or control diet for 21 days. Original magnification, ×10. (**B**) Oil red O staining of the liver neutral lipids in mice, as described in (**A**). Original magnification, ×10. (**C**) Liver weight to body weight of indicated mice. WT-Control, *n* =18, WT-EtOH, *n* = 18, *Elf4^−/−^*-Control, *n* = 18, *Elf4^−/−^*-EtOH, *n* = 10. * *p* < 0.05, **** *p* < 0.0001. Unpaired two-sided Student’s *t*-test, data are represented as mean ± SEM. (**D**–**H**) Triglyceride (TG) level (**D**), alanine aminotransferase (ALT) level (**E**), aspartate transaminase (AST) level (**F**), alkaline phosphatase (ALP) level (**G**), and low-density lipoprotein cholesterol (LDLc) level (**H**) measured from the serum of indicated mice. WT-Control, *n* =18, WT-EtOH, *n* = 18, *Elf4^−/−^*-Control, *n* = 18, *Elf4^−/−^*-EtOH, *n* = 9. NS, not significant, * *p* < 0.05, ** *p* < 0.01, *** *p* < 0.001, **** *p* < 0.0001. Unpaired two-sided Student’s *t*-test, data are represented as mean ± SEM. (**I**) Liver RNA-seq of indicated mice. GO enrichment analysis of genes increased in the *Elf4^−/−^*-EtOH mice compared with the WT-EtOH mice. (**J**) Liver RNA-seq of indicated mice. GO enrichment analysis of genes increased in the *Elf4^−/−^*-Control mice compared with the WT-Control mice.

The hematoxylin and eosin (H&E) staining and Oil red O staining showed that alcohol induced increased hepatic steatosis in *Elf4^−/−^* mice compared to WT mice (Figure 5A,B). Moreover, liver-to-body weight ratio slightly increased in WT mice but significantly increased in *Elf4^−/−^* mice upon alcohol exposure (Figure 5C) (*p* < 0.05 or *p* < 0.0001 as indicated). Furthermore, serum triglyceride (TG) levels had no difference in WT mice, but markedly increased in *Elf4^−/−^* mice (Figure 5D) (*p* < 0.01) as did the alanine aminotransferase (ALT) and aspartate transaminase (AST) levels (Figure 5E,F) (*p* < 0.001 or *p* < 0.05 as indicated). This indicated more severe liver disorders in the *Elf4^−/−^*-EtOH group, compared with the WT-EtOH group. Serum alkaline phosphatase (ALP) and low-density lipoprotein cholesterol (LDLc) levels were decreased in both groups of mice treated with alcohol (Figure 5G,H) (*p* < 0.0001 or *p* < 0.05 as indicated), while serum high-density lipoprotein cholesterol (HDLc), total protein (TP), total cholesterol (TC), and albumin (ALB) levels showed no difference upon alcohol exposure (Supplementary Figure S3A–D). To further explore hepatic metabolic changes, we conducted high-throughput sequencing of liver mRNA (RNA-seq) and compared the gene expressions of *Elf4^−/−^* mice and WT mice upon alcohol exposure. We detected a total of 19,268 genes, of which 568 were upregulated in *Elf4^−/−^* mice and 289 were downregulated in *Elf4^−/−^* mice. Additionally, we conducted GO analysis of any genes which had increased significantly in *Elf4^−/−^* mice. Most of the enriched pathways were mainly correlated with cholesterol metabolism and lipid metabolism (Figure 5I). We selected four pathways (‘cholesterol biosynthetic process pathway’, ‘sterol biosynthetic process pathway’, ‘lipid metabolic process pathway’, and ‘steroid metabolic process pathway’), among which there were 11 overlapping genes which significantly increased in the livers of *Elf4^−/−^* mice, compared with WT mice upon alcohol exposure (Supplementary Figure S3E,F). Taken together, the data revealed that *Elf4^−/−^* mice showed increased alcohol-induced hepatic steatosis.

### 2.6. Alcohol Exposure Increases Hepatic Fibrosis in Elf4^−/−^ Mice

Sirius Red staining of the liver sections showed more Sirius Red positive area in *Elf4^−/−^* mice fed with alcohol diet than in the other three groups (Supplementary Figure S4A). We also detected the mRNA expression of a-SMA which is key indicator of tissue fibrosis and found no difference between the a-SMA mRNA levels in the WT-EtOH group and those of the WT-Control group. However, there was a significant increase in a-SMA in the *Elf4^−/−^*-EtOH group compared with the *Elf4^−/−^*-Control group (Supplementary Figure S4B). These data suggest that *Elf4^−/−^* mice have enhanced hepatic fibrosis.

### 2.7. ELF4 Deficiency Aggravates Alcohol-Induced Increased Permeability

Moreover, we compared the liver gene expression of *Elf4^−/−^* mice and WT mice fed with the control diet. In total, 396 genes showed increased expression in *Elf4^−/−^* mice while 347 genes showed decreased expression in *Elf4^−/−^* mice. The significantly increased genes in *Elf4^−/−^* mice were used for GO analysis and enriched pathways mainly correlated with lipid metabolism and fatty acid metabolism (Figure 5J). This indicates that intestinal ELF4 deficiency can enhance the genes’ expression of lipid metabolism, even without alcohol exposure.

**Figure 6:**
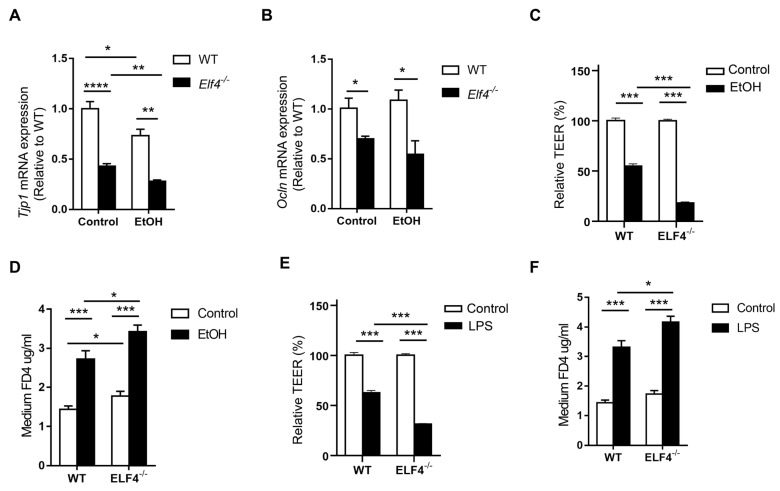
ELF4 deficiency aggravates alcohol-induced increased permeability. (**A**,**B**) Colon *Tjp1* (**A**) and *Ocln* (**B**) mRNA expression of *Elf4^−/−^* mice and WT mice treated with 5% alcohol diet or control diet for 21 days. WT-Control, *n* =10, WT-EtOH, *n* = 14, *Elf4^−/−^*-Control, *n* = 6, *Elf4^−/−^*-EtOH, *n* = 4. * *p* < 0.05, ** *p* < 0.01, **** *p* < 0.0001. Unpaired two-sided Student’s *t*-test, data are represented as mean ± SEM. (**C**,**D**) When TEER was stabilized at around 2000 Ω cm^2^, the WT Caco2 cells and ELF4^−/−^ Caco2 (clone1 was used) cells were treated with ethanol (100 mM) for 6 h, then Caco2 cell permeability to TEER (**C**) and FD4 (**D**) were measured. * *p* < 0.05, *** *p* < 0.001. Unpaired two-sided Student’s *t*-test, data are represented as mean ± SEM. (**E**,**F**) When TEER was stabilized at around 2000 Ω cm^2^, the WT Caco2 cells and ELF4^−/−^ Caco2 (clone1 was used) cells were treated with LPS (100 ng/mL) for 6 h, then Caco2 cell permeability to TEER (**C**) and FD4 (**D**) were measured. Unpaired two-sided Student’s *t*-test, data are represented as mean ± SEM.

To re-determine the mechanism through which ELF4 deficiency leads to increased susceptibility to ALD, we compared the expression of the tight junction genes *Tjp1* and *Ocln*, and the cecal GM composition of *Elf4^−/−^* mice and WT mice fed with the EtOH diet or the control diet. Alcohol feeding decreased *Tjp1* mRNA expression in both the *Elf4^−/−^*-EtOH group and the WT-EtOH group, compared with the *Elf4^−/−^*-Control group and the WT-Control group (Figure 6A) (*p* < 0.05, *p* < 0.01, or *p* < 0.0001 as indicated). Compared with the WT-Control group, the *Tjp1* gene’s expression was significantly decreased in the *Elf4^−/−^*-Control group. This indicated that the loss of ELF4 disrupted the intestinal barrier function. Similarly, the expression of the tight junction protein *Ocln* was significantly decreased in the *Elf4^−/−^*-EtOH group compared with the WT-EtOH group (Figure 6B) (*p* < 0.05).

The effect of ethanol on the transepithelial electrical resistance (TEER) of Caco2 cell monolayers was also evaluated. Compared with WT Caco2 cells, ELF4^−/−^ Caco2 cells took longer to grow into monolayers (Supplementary Figure S5A,B). Ethanol decreased TEER in both WT and ELF4^−/−^ Caco2 cell monolayers, but the decrease was more pronounced in ELF4^−/−^ Caco2 cell monolayers (Figure 6C) (*p* < 0.001). FD4 was used to evaluate the permeability of Caco2 cell monolayers. Compared with WT Caco2 cell monolayers, ELF4^−/−^ Caco2 cell monolayers allowed more FD4 to pass through (Figure 6D) (*p* < 0.05 or *p* < 0.001 as indicated). Ethanol increased the permeability of WT and ELF4^−/−^ Caco2 cell monolayers, and the increase was more pronounced in ELF4^−/−^ Caco2 cell monolayers (Figure 6D). Similarly, LPS can also increase Caco2 cell monolayers’ permeability and ELF4 deletion aggravated the increase (Figure 6E,F) (*p* < 0.05 or *p* < 0.001 as indicated). These data suggest that ELF4 deficiency results in higher permeability and is more sensitive to alcohol-induced increased permeability.

### 2.8. ELF4 Knockout Worsens Alcohol-Induced GM Dysbiosis

**Figure 7:**
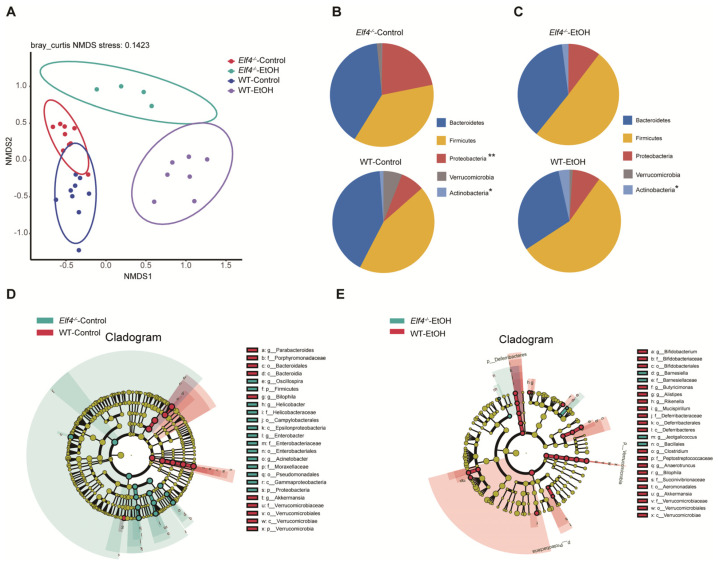
Bacterial 16S rRNA-based analysis of the GM of *Elf4^−/−^* and WT mice. The 6-week-old *Elf4^−/−^* mice and WT mice treated with 5% alcohol diet or control diet for 21 days. Then, mice were sacrificed, cecal contents were taken, and deep sequencing of 16S rRNA was performed. *Elf4^−/−^*-Control mice, *n* = 9, WT-Control mice, *n* = 9, *Elf4^−/−^*-EtOH mice, *n* = 4, WT-EtOH mice, *n* = 7. (**A**) Non-metric multidimensional scaling (NMDS) showed the distance between samples. (**B**) Pie charts showing relative phyla detected in the cecal contents of *Elf4^−/−^*-Control mice and WT-Control mice, 5 most prevalent were shown (* *p* < 0.05, ** *p* < 0.01). (**C**) Pie charts showing relative phylum detected in the cecal contents of *Elf4^−/−^*-EtOH mice and WT-EtOH mice, 5 most prevalent were shown. (**D**) Cladogram showing differential cecal bacteria between *Elf4^−/−^*-Control mice and WT-Control mice with LDA scores greater than 4. (**E**) Cladogram showing differential cecal bacteria between *Elf4^−/−^*-EtOH mice and WT-EtOH mice with LDA scores greater than 2.

Next, we compared the four groups’ (*Elf4^−/−^*-EtOH group, WT-EtOH group, *Elf4^−/−^*-Control group, and WT-Control group) cecal GM compositions. Non-metric multidimensional scaling (NMDS) showed that GM clustered differently between the four groups (Figure 7A). Phyla analysis showed a larger proportion of Firmicutes in the *Elf4^−/−^*-EtOH group and the WT-EtOH group (Figure 7B,C). We also identified that a specific GM was associated with ELF4 deficiency. The *Elf4^−/−^*-Control group had a larger proportion of Proteobacteria (Figure 7B), whereas the *Elf4^−/−^*-EtOH group had a smaller proportion of Actinobacteria (Figure 7C). As shown in Supplementary Figure S6A, the *Elf4^−/−^*-Control group and the WT-Control group had differential genera compositions. The LDA score and cladogram, reflecting relative bacterial abundance (Figure 7D and Supplementary Figure S6B) showed that the *Parabacteroides* genus of the Bacteroidetes phylum, the *Akkermansia* genus of the Verrucomicrobia phylum, and the *Bilophila* genus of the Proteobacteria phylum were overrepresented in the WT-Control group. Conversely, the *Oscillospira* genus of the Firmicutes phylum, the *Enterobacter* genus of the Proteobacteria phylum, the *Helicobacter* genus of the Proteobacteria phylum, and the *Acinetobacter* genus of the Proteobacteria were specifically overrepresented in the *Elf4^−/−^*-Control group. The *Akkermansia* genus of the Verrucomicrobia phylum and the *Bilophila* genus of the Proteobacteria phylum which were specially increased in the WT-Control group, were also overexpressed in the WT-EtOH group (Figure 7E and Supplementary Figure S6C).

## 3. Discussion

Alcohol intake impairs the intestinal barrier and gut homeostasis [6]. An abnormal gut–liver axis, including increased intestinal permeability and gut dysbiosis, contributes to the pathogenesis of ALD [14,16]. However, the mechanisms of intestinal barrier dysfunction are incompletely understood. In the present study, we showed that intestinal ELF4 deletion increased intestinal permeability and gut dysbiosis, altered gut metabolite profiles, and exacerbated liver injury.

Our previous study showed that ELF4 deficiency leads to more severe DNA damage and results in intestinal epithelial cell damage [18]. A recent study has also shown that patients with loss-of-function variants in ELF4 suffered from autoinflammatory disease with IBD characteristics [19]. These findings suggest that ELF4 protects against mucosal disease [18,19]. However, ELF4 deletion’s specific and detailed effects on the gut homeostasis are unknown. Our results suggest that intestinal specific ELF4 deletion disrupts gut homeostasis, causes intestinal barrier dysfunction, and affects the balance of mucosal microbial communities.

Inflammation and TJ barrier dysfunction are intertwined processes [32]. Immune cells infiltrating the intestinal mucosa release proinflammatory cytokines, upregulate the inflammatory cascade and disrupt the intestinal barrier [33,34]. As intestinal ELF4 expression is crucial for maintaining the expression of anti-inflammatory genes [18,19], we postulate that ELF4 deletion disrupts the intestinal barrier. Our results show that *Elf4^−/−^* mice had increased intestinal permeability. Furthermore, we compared the GM compositions and found the gut dysbiosis in *Elf4^−/−^* mice. Gut microbiota composition and metabolites are important factors contributing to the regulation of mucus barrier function [35]. Conversely, intestinal barrier dysfunction leads to gut dysbiosis [13,20]. In our study, the extent of gut barrier disruption was similar among *Elf4^−/−^* mice, regardless of ages (Figure 1A). Our data from cellular experiments showed that ELF4 deletion led to decreased TJ gene expression (Figure 1B) and ELF4^−/−^ Caco2 cell monolayers allowed more FD4 to pass through (Figure 6D). These results suggest that gut barrier disruption occurs before dysbiosis. However, we cannot deny the possibility that ELF4 directly regulates the microbiota. Paneth cells secrete antimicrobial factors and control gut homeostasis [36] and whether ELF4 deletion affects Paneth cells secretion of antimicrobial peptides remains to be further explored.

Our UPLC-MS analysis demonstrated a significant correlation between the differential bacterial members affected by the ELF4 and the differential metabolites. Alterations in metabolic states mediated by gut microbes have been widely used to understand the molecular mechanisms underlying health and disease development in human and animal hosts [37,38].

Alcohol exposure disrupts microbiota homeostasis. Previous studies have shown that alcohol intake decreases the level of Bacteroidetes [4,39], and this is consistent with the microbiota changes in WT mice. Moreover, the *Elf4^−/−^* mice have more severe gut dysbiosis. The *Elf4^−/−^*-Control group had a larger proportion of Proteobacteria, which is regarded as a sign of gut dysbiosis [27]. The GM composition had bigger difference between the *Elf4^−/−^*-Control group and the WT-Control group, meaning that ELF4 deficiency and alcohol have differential influences on GM composition. At the bacterial species level, *Akkermansia* (AKK) and *Bilophila* dropped in the *Elf4^−/−^*-EtOH group, compared with the WT-EtOH group. AKK utilizes mucin as its sole carbon, nitrogen, and energy source [40], and the downregulation of AKK bacteria in *Elf4^−/−^* mice may be due to altered mucin levels due to intestinal injury. AKK is an important probiotic, and its supplementation can ameliorate ALD [40,41]. Recent work has shown that ethanol administration causes a significant increase in *Bilophila* levels [42], but that the role of *Bilophila* in ALD remains unknown.

Shao et al. reported that intestinal hypoxia inducible factor 1α (HIF-1α) is essential for the adaptation response to alcohol exposure-induced changes in intestinal microbiota and barrier function associated with elevated endotoxemia and hepatic steatosis and injury [14], which is similar to our findings. HIF-1α is a master transcription factor regulating a variety of genes in the intestine. Shao et al. emphasized that intestinal *Hif1α^−/−^* mice developed dysbiosis following alcohol exposure, and there was no difference in GM between intestinal *Hif1α^−/−^* mice and WT mice without alcohol exposure [14]. In our study, the *Elf4^−/−^*-Control group had GM dysbiosis with the symptom of a larger proportion of Proteobacteria. At the bacterial species level, Shao et al. reported that AKK was increased in the intestinal *Hif1α^−/−^* mice after alcohol exposure [14], and we found that AKK dropped in the *Elf4^−/−^*-EtOH group, compared with the WT-EtOH group.

To date, most studies with relevance to ALD have focused on the microbiota dysbiosis induced by alcohol [8,14,43]. In our study, we assessed in detail the effects of ELF4 deletion on the gut microbiota and the metabolite profiles. We found that LPS levels were positively correlated with the relative abundance of the Preteobacteria phylum which increased in *Elf4^−/−^* mice. It remains unclear whether gut leakiness and intestinal dysbiosis occur during the early stages of liver injury, or as the result of altered liver function [11]. However, our study provides new evidence of a causal relationship. We compared the liver function and gene expression between the *Elf4^−/−^*-Control group and the WT-Control group. The AST and ALT levels showed no difference between the *Elf4^−/−^*-Control group and the WT-Control group. Though the liver function showed no obvious difference, many genes’ expressions were correlated with the lipid metabolism and fatty acid metabolism enriched in *Elf4^−/−^* mice. *Elf4^−/−^* mice have an abnormal metabolite profile with a significant increase in LPS levels. Previous studies have shown that LPS induces liver inflammation through TLR4, resulting in metabolic perturbations [9,44]. The intestinal ELF4 deficiency leads to dysregulation of liver gene expression, which makes the liver vulnerable and more prone to ALD in response to alcohol stimulation.

In conclusion, our findings demonstrate that intestinal epithelial ELF4 is critical to the prevention of ALD through the maintenance of the intestinal barrier function and gut homeostasis. Intestinal ELF4 deficiency resulted in GM dysbiosis with increased Proteobacteria proportion and LPS level. Alcohol exposure leads to more severe hepatic inflammation and steatosis in *Elf4^−/−^* mice. Thus, we identify ELF4 as an important host protective factor in maintaining gut homeostasis and alleviating alcohol exposure-induced hepatic steatosis and injury, and our study improves the understanding of ALD’s pathogenesis. Our study suggests that ELF4 can be used as a potential therapeutic target for ALD, and drugs or diets that can effectively upregulate intestinal ELF4 can be developed to prevent and treat ALD. There are also limitations of this study. We cannot rule out the possibility that ELF4 directly affects antimicrobial peptides leading GM dysbiosis. Additionally, the therapeutic role of ELF4 in ALD is limited due to the lack of an agonist of ELF4.

## 4. Materials and Methods

### 4.1. Mice and ALD (Alcohol-Related Liver Disease) Induction

Intestinal epithelial specific ELF4 knockout (*Elf4^−/−^*) mice were generated by crossing Elf4^loxp/loxp^ mice which have been described previously [18] and a transgenic strain expressing Cre recombinase under the control of the murine villin promoter (T000142, Model Animal Research Center of Nanjing University, Nanjing, China). All animals were maintained in a SPF (special pathogen-free) environment under 12 h light–dark cycles with access to food and water ad libitum.

Six-week-old male mice (WT (Cre mice) and *Elf4^−/−^*) were pair-fed the Lieber–DeCarli liquid diet containing 5% (*w*/*v*) alcohol (EtOH) or isocaloric maltose dextrin (Control) for 21 days; body weight and survival were recorded.

### 4.2. Intestinal Permeability Test

Both male and female mice were used. Fasting sex- and age-matched WT mice and *Elf4^−/−^* mice for 4 h, 4 kDa FITC-Dextran (FD4) was given by gavage (100 mg/kg, 4013, Chondrex, Woodinville, WA, USA). Then, 3 h later, blood was taken from the inner canthus of mice and allowed to stand at room temperature for two hours, then centrifuged at 4000 rpm for 15 min in a low-temperature centrifuge. The serums’ OD530 were recorded and the serum FD4 concentration was calculated according to the standard curve.

### 4.3. CRISPR-Mediated ELF4 Knockout Cell Line

In vitro experiments were performed using Caco2 cell line. To generate ELF4^−/−^ Caco2 cells, we designed sgRNAs targeting the human ELF4 exon 3 coding sequences, and the sgRNAs were cloned into the inducible lentiviral vector (lentiGuide-Puro). Lentivirus packaging, transduction, and generation of ELF4 knockout stable cell lines were performed as previously described [18].

### 4.4. Whole Genome RNA Sequencing and GSEA Analysis

For mice, WT mice and *Elf4^−/−^* mice were fed with a liquid diet containing 5% (*w*/*v*) alcohol or isocaloric maltose dextrin for 21 days. On the last day, mice were sacrificed, and liver mRNA was isolated with Trizol reagent (Invitrogen, Carlsbad, CA, USA). For cells, the mRNA of ELF4^−/−^ Caco2 cells and WT Caco2 cells was isolated with Trizol reagent. The transcriptome library Illumina^®^ (Vazyme Biotech Co.,Ltd, Nanjing, China) was used following the manufacturer’s recommendations. The libraries were then sequenced on the Illumina Hiseq platform using (2 × 150 bp) paired-end module. The raw reads in fastq format passed FastQC quality control [45]. Then, the raw reads in fastq format were mapped to hg38 (for human cell line) or mm10 (for mice), and sam format files output via HISAT2 [46]. Samtools was used to convert these sam format files to bam format files, and resort these bam format files [47]. Then, the gene reads were counted by HTSeq [48] and analyzed with DEseq2 [49].

For GSEA analysis, the package was downloaded from the official website (https://www.gsea-msigdb.org/gsea/index.jsp accessed on 12 August 2021). The data was prepared as recommended and the c5.bp.v7.1.symbols.gmt file was used as gene sets.

### 4.5. 16S rRNA NovaSeq Sequencing and Microbiota Analysis

Cecal feces were collected from WT mice and *Elf4^−/−^* mice. The genome DNA was extracted using the CTAB method. 16s rRNA genes of distinct regions (16S V3-V4) were amplified using the specific primer (341F: 5′-CCTAYGGGRBGCASCAG-3′ and 806R: 5′-GGACTACNNGGGTATCTAAT-3′) with the barcode. Sequencing libraries were generated using TruSeq^®^ DNA PCR-Free Sample Preparation Kit (Illumina, San Diego, CA, USA) following the manufacturer’s recommendations and index codes were added. The library quality was assessed on the Qubit@ 2.0 Fluorometer (Thermo Fisher Scientific, Waltham, MA, USA). At last, the library was sequenced on an Illumina NovaSeq platform and 250 bp paired-end reads were generated. The analysis was conducted by following the “Atacama soil microbiome tutorial” of Qiime2docs along with customized program scripts (https://docs.qiime2.org/2019.1/ accessed on 20 June 2021). Briefly, raw data fastq files from each sample were quality filtered and trimmed, de-noised, merged, and then the chimeric sequences were identified and removed using QIIME2 to obtain the feature table of amplicon sequence variant (ASV) [50]. The ASV sequences were aligned to a pre-trained GREENGENES 13_8 99% database (trimmed to the V3V4 region bound by the 338F/806R primer pair) to generate the taxonomy table. LEfSe was employed to identify the bacteria with different abundance among samples and groups [51].

### 4.6. Untargeted Metabolomics

Metabolite extraction was performed according to the method reported in the previous article [52]. Feces (100 mg) from *Elf4^−/−^* mice and WT mice were individually grounded with liquid nitrogen and the homogenate was resuspended with prechilled 80% methanol by well vortex. The samples were incubated on ice for 5 min and then were centrifuged at 15,000× *g*, 4 °C for 20 min. Some of supernatant was diluted to final concentration containing 53% methanol by LC-MS grade water. The samples were subsequently transferred to a fresh Eppendorf tube and then were centrifuged at 15,000× *g*, 4 °C for 20 min. Finally, the supernatant was injected into the LC-MS/MS system analysis.

UHPLC-MS/MS analyses were performed using a Vanquish UHPLC system (Thermo Fisher Scientific, Waltham, MA, USA) coupled with an Orbitrap Q ExactiveTM HF mass spectrometer. Samples were injected onto a Hypesil Goldcolumn (100 × 2.1 mm, 1.9 μm) using a 17 min linear gradient at a flow rate of 0.2 mL/min. The eluents for the positive polarity mode were eluent A (0.1% FA in Water) and eluent B (Methanol). The eluents for the negative polarity mode were eluent A (5 mM ammonium acetate, pH 9.0) and eluent B (Methanol). The solvent gradient was set as follows: 2% B, 1.5 min; 2–100% B, 12.0 min; 100% B, 14.0 min; 100–2% B, 14.1 min; 2% B, 17 min. The Q ExactiveTM HF mass spectrometer was operated in positive/negative polarity mode with spray voltage of 3.2 kV, capillary temperature of 320 °C, sheath gas flow rate of 40 arb, and aux gasflow rate of 10 arb.

The raw data files generated by UHPLC-MS/MS were processed using the Compound Discoverer 3.1 (CD3.1, ThermoFisher, Waltham, MA, USA), and peak alignment, peak picking, and quantitation were performed for each metabolite. The main parameters were set as follows: retention time tolerance, 0.2 min; actual mass tolerance, 5 ppm; signal intensity tolerance, 30%; signal/noise ratio, 3; etc. After that, peak intensities were normalized to the total spectral intensity. The normalized data was used to predict the molecular formula based on additive ions, molecular ion peaks, and fragment ions. Then, peaks were matched with the mzCloud (https://www.mzcloud.org/ accessed on 21 September 2021), mzVault, and MassList databases to obtain the accurate qualitative and relative quantitative results.

These metabolites were annotated using the KEGG database (https://www.genome.jp/kegg/pathway.html accessed on 21 September 2021), HMDB database (https://hmdb.ca/metabolites accessed on 21 September 2021), and LIPIDMaps database (http://www.lipidmaps.org/ accessed on 21 September 2021). Data normalization, principal components analysis (PCA), orthogonal partial least squares discriminant analysis (OPLS-DA), and random forest (RF) were performed with R package MetaboAnalystR [53]. To make the data close to a normal distribution, the Normalization function in MetaboAnalystR package was adopted. We applied a univariate analysis (*t*-test) to calculate the statistical significance (*p*-value). The *p*-value < 0.05 and log2(Fold Change) > 1.8 were considered indicative of differential metabolites. A volcano plot was used to filter metabolites of interest based on log2(Fold Change) and −log10(p-value), which was conducted using ggplot2 package in R language.

### 4.7. Histopathological Analysis

Liver tissues were fixed in 10% formalin, then imbedded in paraffin. The 5 μm sections of these livers were stained with hematoxylin–eosin (H&E) following the standard protocol and analyzed by light microscopy. Liver sections were stained with Oil red O stain to visualize neutral lipids and Sirius red stain to visualize liver fibrosis.

### 4.8. Biochemical Analysis and Cytokine Measurements

Serum Triglyceride (TG), alanine aminotransferase (ALT), aspartate transaminase (AST), alkaline phosphatase (ALP), low-density lipoprotein cholesterol (LDLc), high-density lipoprotein cholesterol (HDLc), total protein (TP), total cholesterol (TC), and albumin (ALB) level were analyzed with an automatic blood biochemical analyzer (Mindary, BS-180). IL6 (RK0008), TNFa (RK0027), and IL10 (RK0016) were measured from plasma and liver cells’ lysate by ELISA according to manufacturer’s recommendations.

### 4.9. Transepithelial Electrical Resistance (TEER) Assay

Cells were inoculated in cell culture transwell (Corning, NY, USA) with a membrane area of 1.12 cm^2^ and a pore size of 0.4 μm and placed in 12-well plates. Cells were ready for study when the transepithelial electrical resistance (TEER) was stable (~2000 Ωcm^2^). TEER was measured using a milli-cell resistance system (Merck-Milibert, Darmstadt, Germany). After the TEER value was determined for the last time, 100 μL FD4 (1 mg/mL) was added to the transwell insert (Costar Upper Compartment, Corning, NY, USA). Petri dishes were incubated at 37 °C for 30 min, then 100 μL of medium was collected from each plate well (lower) to detect the fluorescence intensity at an excitation wavelength of 480 nm and an emission wavelength of 520 nm.

### 4.10. Quantitative PCR (qPCR)

Total RNA form liver and colon tissues was extracted with Trizol reagent (Invitrogen) and then reverse transcribed (RT) into cDNA according to the manufacturer’s protocol. The cDNA was amplified with a SYBR green PCR mix (Vazyme, R233-01) on the ABI 7500 Detection System (all primers, Table 1). Data were represented as relative mRNA levels using the ΔΔCT method.

### 4.11. Quantitative Proteomics

WT and ELF4^−/−^ Caco2 cells were homogenized in co-immunoprecipitation lysis buffer for total protein preparation. In total, 50ug of protein from each sample was subjected to mass spectrometry analysis. Mass spectrometry data were acquired with an LTQ Orbitrap Elite mass spectrometer (Thermo Fisher Scientific, Waltham, MA, USA).

### 4.12. Statistical Analysis

Samples were compared using two-tailed, unpaired Student’s *t*-test, unless otherwise stated with GraphPad Prism 7.00. Error bars were represented by SEM.

## Figures and Tables

**Table 1:** Primers used in qPCR.

Gene	Forward Primer (5′-3′)	Reverse Primer (5′-3′)
Murine Ocln	TGAAAGTCCACCTCCTTACAGA	CCGGATAAAAAGAGTACGCTGG
Murine Tjp1	GCCGCTAAGAGCACAGCAA	GCCCTCCTTTTAACACATCAGA
Murine a-SMA	CCCAGACATCAGGGAGTAATGG	TCTATCGGATACTTCAGCGTCA
Murine Tnfα	CCCCAAAGGGATGAGAAGTT	TGGGCTACAGGCTTGTCACT
Murine Ccl2	GGGCCTGCTGTTCACAGTT	GGGATCATCTTGCTGGTGAA
Murine Il1b	TGCACGCTCCGGGACTCACA	CATGGAGAACACCACTTGTTGCTCC
Murine Hprt	TCAGTCAACGGGGGACATAAA	GGGGCTGTACTGCTTAACCAG
Murine Gapdh	ATTCAACGGCACAGTCAAGG	GCAGAAGGGGCGGAGATGA
Human βACTIN	TCCCTGGAGAAGAGCTACG	GTAGTTTCGTGGATGCCACA
Human OCLN	ACAAGCGGTTTTATCCAGAGTC	GTCATCCACAGGCGAAGTTAAT

## Data Availability

The RNA-seq data that support the findings in this study have been deposited in the GEO database with the accession code GSE192924. Raw data from Figures were deposited on Mendeley Data (doi:10.17632/58w2wx93xk.1).

## Supplementary Materials

The following supporting information can be downloaded at: https://www.mdpi.com/article/10.3390/ijms23094825/s1.
