# Supplementary material for: Intestinal ELF4 Deletion Exacerbates Alcoholic Liver Disease by Disrupting Gut Homeostasis
Source: Int J Mol Sci. 2022 Apr 27;23(9):4825. doi: 10.3390/ijms23094825 (PMC9102452; doi:10.3390/ijms23094825)
Supplement: Supplementary file 1 [file ijms-23-04825-s001.zip › ijms-1663251-supplementary.pdf]

Supplementary Figure S1 Information of *Elf4*$^{-/-}$ mice and ELF4$^{-/-}$ Caco2 cells.

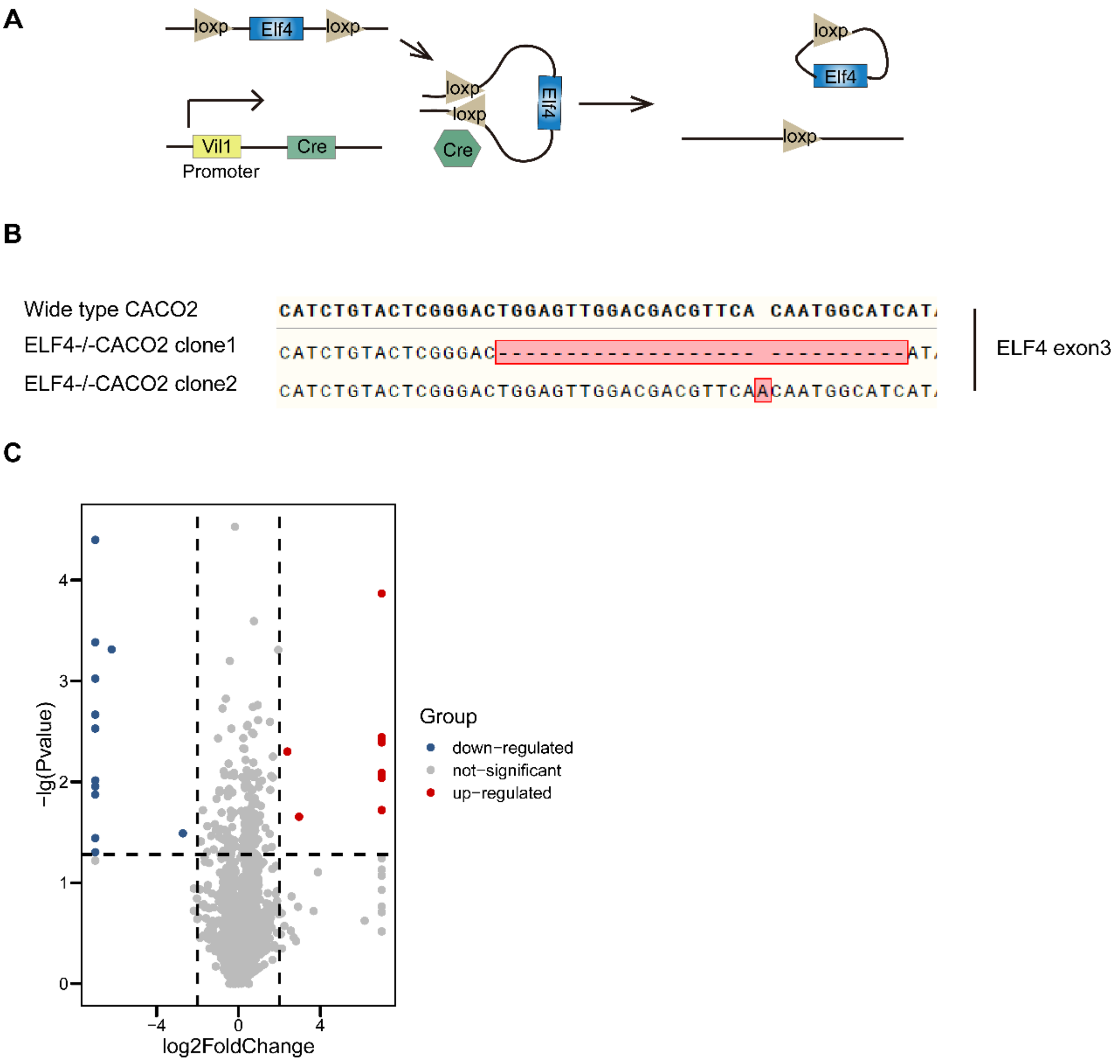

(**A**) Diagram of generation of intestinal specific *Elf4* knockout mice. (**B**) The diagram of two ELF4$^{-/-}$ Caco2 clones. Clone 1 has a 16 bp deletion in ELF4 exon3, and clone2 has 1 bp insertion in ELF4 exon3. (**C**) Volcano plot of protein expression in ELF4$^{-/-}$ Caco2 cells compared with WT Caco2 cells. The log2FoldChange of proteins which were only detected in ELF4$^{-/-}$ Caco2 cells were recorded as 7, and the log2FoldChange of proteins only detected in WT Caco2 cells were recorded as -7.

Supplementary Figure S2 Permutation test for OPLS-DA models.

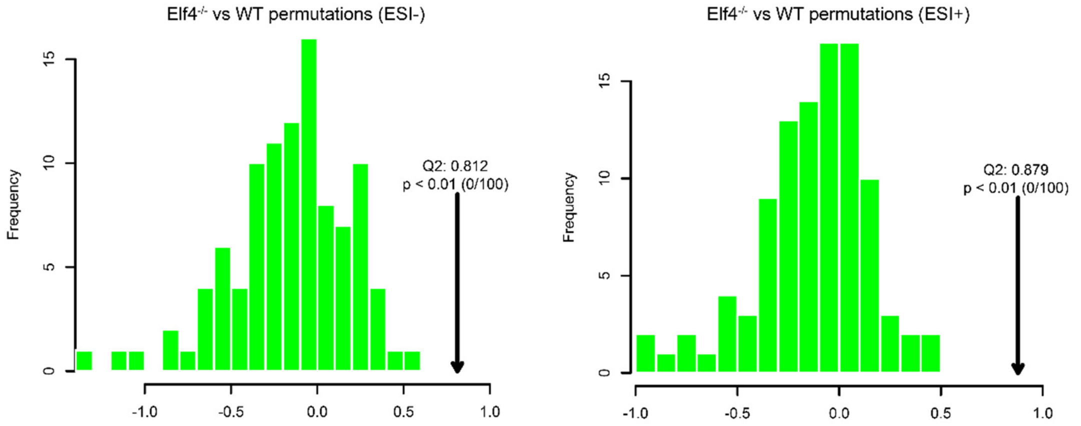

Permutation test for OPLS-DA models between Elf4$^{-/-}$ group and WT group in ESI- model (left panel) and ESI+ model (right panel).

Supplementary Figure S3 *Elf4$^{-/-}$* mice have increased genes expression of lipid and sterol metabolism.

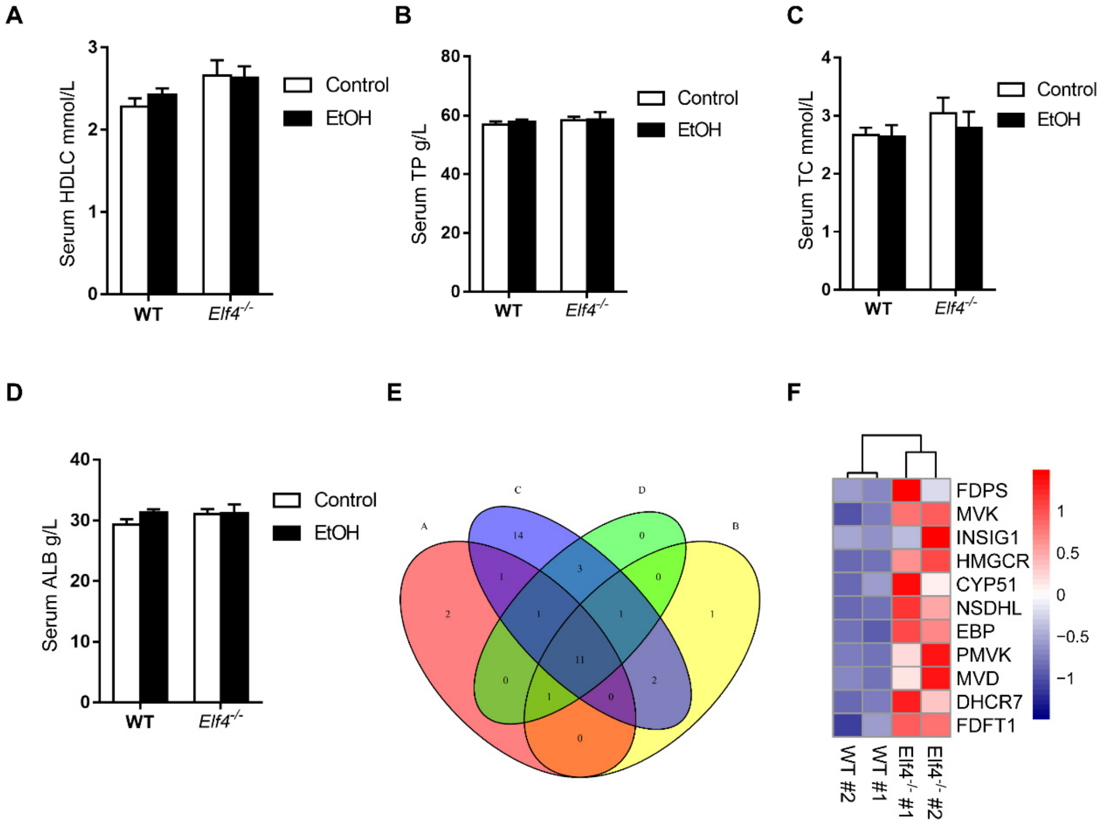

(**A-D**) High-density lipoprotein cholesterol (HDLC) level (**A**), total protein (TP) level (**B**), total cholesterol (TC) level (**C**) and albumin (ALB) level (**D**) measured form serum of indicated mice. WT-Control, n =18, WT-EtOH, n=18, *Elf4*$^{-/-}$-Control, n=18, *Elf4*$^{-/-}$-EtOH, n=9. Data are represented as mean±SEM. (**E**) Venn diagram showing the overlapping genes between GO pathways enriched in the liver RNA-seq of *Elf4*$^{-/-}$-EtOH mice compared with WT-EtOH mice. 'A' refers to cholesterol biosynthetic process pathway, 'B' refers to sterol biosynthetic process pathway, 'C' refers to lipid metabolic process pathway and 'D' refers to steroid metabolic process pathway. (**F**) Heatmap of overlapping genes mRNA expression of four pathways, described as in **E**.

Supplementary Figure S4 *Elf4*$^{-/-}$ mice have exacerbated liver fibrosis by alcohol treatment.

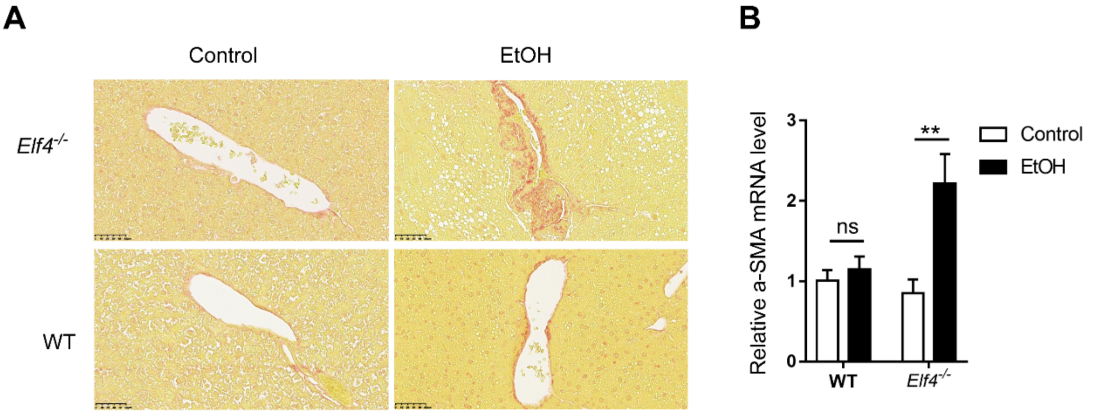

(**A**) Sirius Red staining of the liver sections from *Elf4*$^{-/-}$ mice and WT mice treated with alcohol diet or control diet for 21 days. Original magnification, ×40. (**B**) a-SMA gene expression from the livers of mice described as in A, WT-Control, n =7, WT-EtOH, n=12, *Elf4*$^{-/-}$-Control, n=8, *Elf4*$^{-/-}$-EtOH, n=4. NS, not significant, **P=0.0027. Unpaired two-sided Student' s t-test, data are represented as mean±SEM.

Supplementary Figure S5 TEER of Caco2 cells.

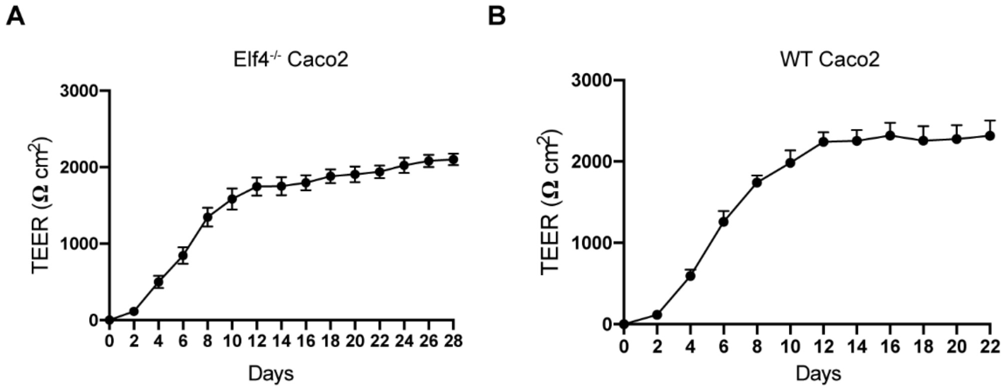

(**A-B**) Change in the TEER value of WT Caco2 cells and ELF4$^{-/-}$ Caco2 cells over time. The TEER was determined every 2 days. When the resistance approached 2000 Ω cm2, the monolayer ELF4$^{-/-}$ Caco2 cells (**A**) and WT Caco2 cells (**B**) were used in the current trail.

Supplementary Figure S6 Bacterial 16S rRNA-based analysis of the GM of *Elf4*$^{-/-}$ and WT mice.

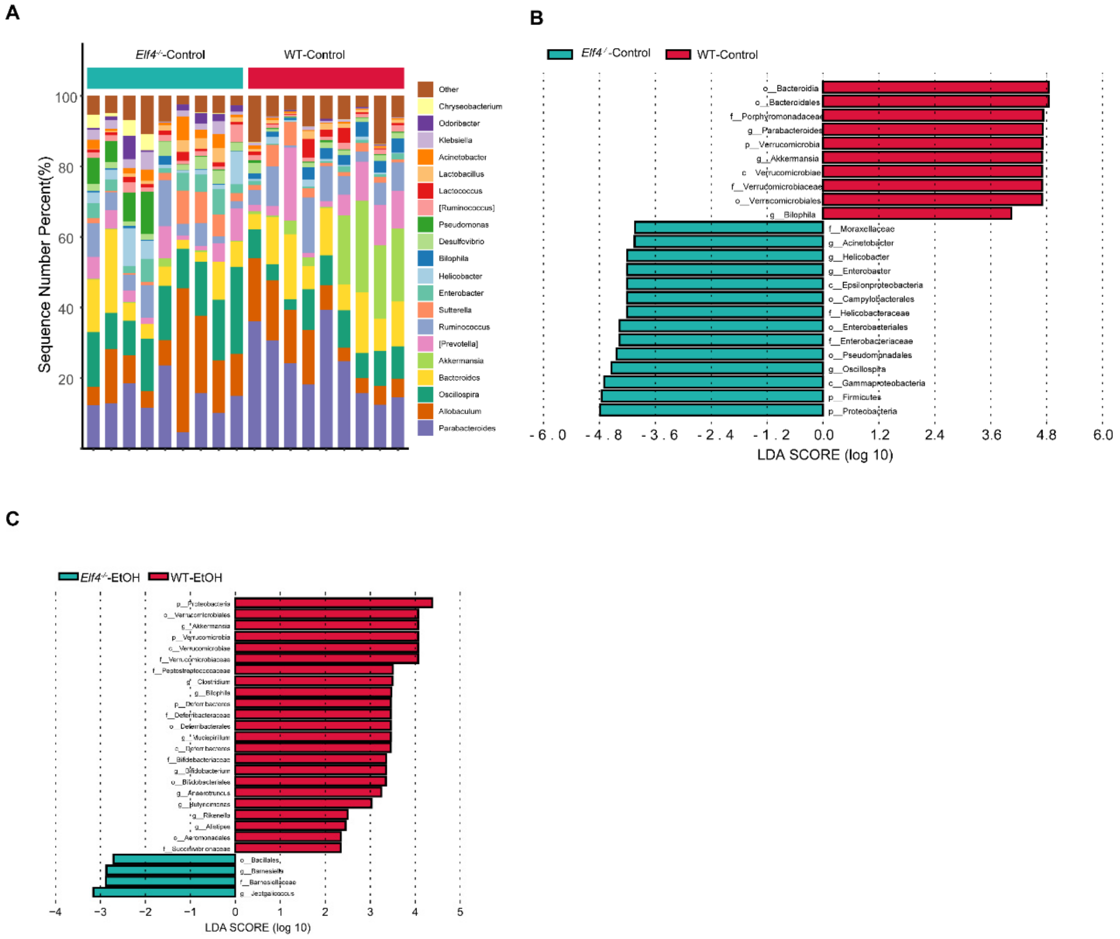

(**A**) Sequence number percentage for various intestinal bacteria genera in the *Elf4*$^{-/-}$-Control mice and the WT-Control mice samples, top 20 genera were shown. (**B**) Linear discriminant analysis Effect Size (LEfSe) analysis showing the differential fecal bacteria genera between *Elf4*$^{-/-}$-Control mice and WT-Control mice with linear discriminant analysis (LDA) scores greater than 4. *Elf4*$^{-/-}$-Control, n=9, WT-Control, n=9. (**C**) Linear discriminant analysis Effect Size (LEfSe) analysis show the differential fecal bacteria genera between *Elf4*$^{-/-}$-EtOH mice and WT-EtOH mice with linear discriminant analysis (LDA) scores greater than 2. *Elf4*$^{-/-}$-EtOH, n=4, WT-EtOH, n=4.
